# RhoG Protein Regulates Platelet Granule Secretion and Thrombus Formation in Mice*[Fn FN2]

**DOI:** 10.1074/jbc.M113.504100

**Published:** 2013-10-08

**Authors:** Robert Goggs, Matthew T. Harper, Robert J. Pope, Joshua S. Savage, Christopher M. Williams, Stuart J. Mundell, Kate J. Heesom, Mark Bass, Harry Mellor, Alastair W. Poole

**Affiliations:** From the ‡ School of Physiology and Pharmacology,; § Proteomics Facility, and; ¶ School of Biochemistry, University of Bristol, Bristol BS8 1TD, United Kingdom

**Keywords:** Platelets, Proteomics, Rho GTPases, Secretion, Thrombosis, Transgenic Mice

## Abstract

Rho GTPases such as Rac, RhoA, and Cdc42 are vital for normal platelet function, but the role of RhoG in platelets has not been studied. In other cells, RhoG orchestrates processes integral to platelet function, including actin cytoskeletal rearrangement and membrane trafficking. We therefore hypothesized that RhoG would play a critical role in platelets. Here, we show that RhoG is expressed in human and mouse platelets and is activated by both collagen-related peptide (CRP) and thrombin stimulation. We used RhoG^−/−^ mice to study the function of RhoG in platelets. Integrin activation and aggregation were reduced in RhoG^−/−^ platelets stimulated by CRP, but responses to thrombin were normal. The central defect in RhoG^−/−^ platelets was reduced secretion from α-granules, dense granules, and lysosomes following CRP stimulation. The integrin activation and aggregation defects could be rescued by ADP co-stimulation, indicating that they are a consequence of diminished dense granule secretion. Defective dense granule secretion in RhoG^−/−^ platelets limited recruitment of additional platelets to growing thrombi in flowing blood *in vitro* and translated into reduced thrombus formation *in vivo*. Interestingly, tail bleeding times were normal in RhoG^−/−^ mice, suggesting that the functions of RhoG in platelets are particularly relevant to thrombotic disorders.

## Teaser

**Background:** Rho family GTPases are essential for normal platelet function, but the role of RhoG in platelets has not been studied previously.

**Results:** Platelets lacking RhoG have reduced granule secretion, and RhoG null mice have impaired thrombus formation.

**Conclusion:** RhoG is required for normal platelet granule secretion.

**Significance:** RhoG is a novel regulator of platelet function and thrombosis.

## Introduction

Platelets are key to the initial response to vascular endothelial damage and are integral to the cell-based model of hemostasis (1, 2). At sites of atherosclerotic plaque rupture, accumulation of activated platelets leads to thrombosis, coronary occlusion, and myocardial infarction (3), and it is likely platelets are also involved in the pathogenesis of atherosclerosis itself (4). Central to these phenomena are the secretory functions of platelets (5). Under high-shear conditions in stenotic arteries, positive autocrine and paracrine feedback mechanisms augment and stabilize platelet-rich thrombi. Secretion of ADP from platelet dense granules and consequent activation of the platelet P2Y_1_ and P2Y_12_ receptors are required for thrombus growth and stability (6, 7). In addition to soluble platelet agonists from dense granules, platelets release coagulation factors, adhesion molecules, growth factors, and chemokines from their α-granules (8), which not only facilitate the platelets' primary hemostatic functions but also contribute to a wide range of pathophysiological processes, including atherogenesis, inflammation (4), asthma (9), and cancer metastasis (10).

On encountering subendothelial collagen or thrombin generated at sites of vessel injury, platelets become activated through multiple signaling pathways (11), leading to cytoskeletal rearrangement, filopodial extension, and granule secretion. The activity of Rho family GTPase proteins underpins these functional events (12), and Rac (13), RhoA (14), and Cdc42 (15) have established roles as regulators of platelet function. Intriguingly, in addition to these three GTPases, transcript and proteomics studies suggest that human platelets also express other Rho family members, including RhoB, RhoC, Rif (RhoF), RhoG, RhoH, and RhoQ (16, 17), but the functions of these other GTPases in platelets are unknown.

We were particularly interested in the role of RhoG in platelets. In other cells, RhoG regulates the actin cytoskeleton and is involved in formation of filopodia (18) and lamellipodia (19) and in integrin-mediated spreading (20), with this regulation frequently occurring through Rac1 or Cdc42 (21, 22). RhoG also regulates the membrane dynamics involved in macropinocytosis (23) and the microtubule rearrangements necessary for lysosomal trafficking (24). Given the importance of actin cytoskeletal rearrangements, microtubule dynamics, and granule trafficking to platelet function, we hypothesized that platelets lacking RhoG would be dysfunctional, leading to defective hemostasis in RhoG^−/−^ mice.

In this study, we combined RhoG activation assays, pharmacologic approaches, proteomics, and a RhoG null mouse line to examine the roles and regulation of RhoG in platelets. We found that in both human and mouse platelets, RhoG is activated by stimulation with thrombin or with the glycoprotein (GP)^3^ VI-specific agonist collagen-related peptide (CRP) and that this activation is dependent on the activity of Src family kinases and Syk. We show that integrin activation, aggregation, cell adhesion, and *in vitro* thrombus formation are all reduced downstream of GPVI in RhoG^−/−^ platelets. In seeking to explain these defects, we identified interactions between active RhoG and regulators of the actin cytoskeleton and secretion. Following CRP stimulation, RhoG operates independently of Rac to control platelet secretion from α-granules, dense granules, and lysosomes. Secretion in RhoG^−/−^ platelets is normal following thrombin stimulation. We propose that reduced dense granule secretion is the central abnormality in RhoG^−/−^ platelets because function can be “rescued” by co-stimulation with ADP. Most importantly, defective platelet function in RhoG^−/−^ mice translates into a reduction in thrombus formation *in vivo*, without adversely affecting hemostatic potential. This work demonstrates novel interactions for RhoG and establishes RhoG as an important regulator of platelet function with potential relevance to the roles of platelets in disease.

## EXPERIMENTAL PROCEDURES

### 

#### 

##### Materials

Except where specified, all chemicals were obtained from Sigma. NuPAGE lithium dodecyl sulfate sample buffer was from Invitrogen. Antibodies against DOCK1, ELMO1, RhoA, RhoG, LAT, SLP76, and VAMP2 were from Santa Cruz Biotechnology (via Insight Biotechnology, Wembley, United Kingdom). Anti-VAMP8 antibodies were from Abcam (Cambridge, United Kingdom). Antibodies against Rac, Cdc42, Src, PKCα, and PI3K p85; phosphospecific antibodies against Src, Syk, LAT, phospholipase Cγ2 (PLCγ2), and PI3K p85; and PKC substrates were from Cell Signaling Technology (via New England Biolabs, Hitchin, United Kingdom). Anti-Syk and anti-phospho-SLP76 antibodies were from BD Biosciences. HRP-conjugated secondary antibodies, ECL solutions, and autoradiography film were from Amersham Biosciences. Western blotting solutions and equipment were from Bio-Rad and Fermentas (St. Leon-Rot, Germany). Phycoerythrin-conjugated anti-active α_IIb_β_3_ antibody (JON/A); DyLight 488-conjugated anti-GPIbβ antibody; and FITC-conjugated anti-GPVI, anti-GPIbα, anti-GPIa, and anti-CD62P antibodies were from Emfret Analytics (Würzburg, Germany). FITC-conjugated anti-CD41 and anti-rat IgG1 antibodies were from AbD Serotec (Kidlington, United Kingdom). AR-C66096, BAPTA-AM, BIM1, MRS-2279, piceatannol, Su6656, U73122, and wortmannin were from Tocris (via R&D Systems, Oxford, United Kingdom). Abciximab was from Eli Lilly. Luciferin-luciferase (Chrono-Lume reagent) and Horm collagen (Chrono-Par collagen) were from Chrono-Log (via Labmedics, Manchester, United Kingdom). CRP was synthesized by Prof. Richard Farndale (University of Cambridge, Cambridge, United Kingdom). 3,3′-Dihexyloxacarbocyanine iodide was from Axxora (Nottingham, United Kingdom). Mowiol and d-phenylalanyl-prolyl-arginyl chloromethyl ketone were from Calbiochem, and PVDF membrane was from Millipore (all via Merck Chemicals).

##### Animals

RhoG^−/−^ mice, as reported previously (25), were a gift from Dr. Len Stephens (Babraham Institute, Cambridgeshire, United Kingdom). Age-matched RhoG^+/+^ littermates (henceforth known as wild-type) were used as controls. Mice were bred and maintained in the University of Bristol animal facilities according to United Kingdom Home Office regulations. All procedures undertaken complied with the Animals (Scientific Procedures) Act 1986 (licenses 30/2386 and 30/2908). The University of Bristol Local Research Ethics Committee approved use of mice for this study.

##### Platelet Preparation

8–16-week-old mice of mixed gender were used. Blood was drawn into 4% trisodium citrate (1:9) by descending vena cava puncture of mice humanely killed by rising CO_2_ inhalation per Animals (Scientific Procedures) Act Schedule 1 (1986). Prior to platelet preparation, complete blood counts were conducted (Pentra ES 60, Horiba) and adjusted for anticoagulant volume. Washed mouse platelets were prepared as described previously (26).

Human blood was obtained from healthy drug-free volunteers in accordance with Local Research Ethics Committee-approved guidelines (E5736). Written informed consent was obtained in accordance with the Declaration of Helsinki. Indomethacin (10 μm) and apyrase (0.02 units/ml) and prostaglandin E_1_ (140 nm) were added to platelet-rich plasma as described previously (27). All platelets were resuspended in Tyrode's HEPES buffer (135 mm NaCl, 3 mm KCl, 10 mm HEPES, 5 mm glucose, and 1 mm MgCl_2_·6H_2_O, pH 7.3), counted using a Z1 Coulter Counter (Beckman Coulter), diluted as required, and rested for 30 min at 30 °C in the presence of indomethacin and apyrase before use.

##### GST-RhoG Pulldown Proteomics

pGEX plasmids containing the sequence for RhoG and GST-RhoG fusion proteins were made using standard techniques. GST-RhoG-Sepharose beads were washed with exchange buffer (20 mm HEPES, pH 7.5, 10 mm EDTA, 100 mm NaCl, and 1 mm dithiothreitol) and then loaded with 1 mm GDP or 100 μm GTPγS (final concentrations) for 30 min at 4 °C with rotation. The nucleotide exchange reaction was terminated by the addition of MgCl_2_ to a final concentration of 60 mm. Nucleotide-loaded GST-RhoG beads (or equal volumes of GSH-Sepharose beads only) were then rotated with washed platelet lysates (500 μl, 5 × 10^8^/ml) for 1 h at 4 °C. Beads were recovered by centrifugation, and unbound proteins were removed by repeated washing. Bound proteins were eluted in 2× Laemmli buffer by boiling at 98 °C for 5 min, and samples were then processed for mass spectrometry or for immunoblotting.

Proteomics was performed as described previously (28) with modifications. Proteins were fractionated by SDS-PAGE. Gel lanes were sectioned and subjected to in-gel tryptic digestion, and the resulting peptides were fractionated by HPLC. Peptides were ionized by nanoelectrospray, and tandem mass spectra were acquired using a mass spectrometer (LTQ Orbitrap Velos, Thermo Scientific) operated in data-dependent acquisition mode. The spectrometer was set to analyze the survey scans at 60,000 resolution (*m*/*z* 400) at *m*/*z* 300–2000. The top 20 multiply charged ions in each duty cycle were selected for MS/MS in the LTQ linear ion trap. Charge state filtering, where unassigned precursor ions were not selected for fragmentation, and dynamic exclusion (repeat count of 1, repeat duration of 30 s, and exclusion list size of 500) were used. Fragmentation conditions in the LTQ were as follows: normalized collision energy of 40%, activation *q* of 0.25, activation time of 10 ms, and minimum ion selection intensity of 500 counts. Raw data files acquired using Xcalibur v2.1 software were processed and quantified using Proteome Discoverer v1.2 software (both from Thermo Scientific) and searched against UniProt/Swiss-Prot human database release version 57.3 (20,326 entries) using the SEQUEST (v28.13) algorithm. Peptide precursor mass tolerance was set at 10 ppm, and MS/MS tolerance was set at 0.8 Da. Search criteria included carbamidomethylation of cysteine (+57.0214) as a fixed modification and oxidation of methionine (+15.9949) as a variable modification. Searches were performed with full tryptic digestion, and a maximum of 1 missed cleavage was allowed. The reverse database search option was enabled, and peptide data were filtered to satisfy a 5% false discovery rate. Databases were then manually reviewed to remove contaminant proteins such as keratin, plasma proteins, and proteins from cells other than platelets. UniProt/Swiss-Prot database accessions were checked, and where necessary, BLASTp searches were performed to confirm the identity of the peptides. All original and subsequently refined lists are presented.

##### Immunoblotting

Washed platelets (4 × 10^8^/ml) stimulated as indicated were lysed in Laemmli buffer containing 50 mm dithiothreitol. Proteins were separated by electrophoresis using 8–15% Tris glycine-polyacrylamide gels against known molecular weight markers and transferred onto PVDF membranes. After blocking with 5% BSA in Tris-buffered saline/Tween-20 (10 mm Tris, 150 mm NaCl, and 0.1% Tween 20), membranes were probed with the appropriate primary and horseradish peroxidase-conjugated secondary antibodies, and proteins were detected by enhanced chemiluminescence.

##### RhoG Activation Assay

The pGEX plasmid containing the sequence for ELMO2 (amino acids 1–362) was a gift from Dr. H. Katoh (Kyoto University, Kyoto Japan). GST-ELMO fusion proteins bound to GSH-Sepharose were prepared by standard techniques. Washed platelet aliquots (500 μl, 5 × 10^8^/ml) stimulated as indicated (20 °C) were lysed on ice with equal volumes of 2× lysis buffer (0.1 m Tris-Cl, 1 m NaCl, 20 mm MgCl_2_, 2% Triton X-100, and EDTA-free protease inhibitors). GST-ELMO beads were rotated with platelet lysates for 1 h at 4 °C, and unbound proteins were removed by washing. Bound proteins were eluted in Laemmli buffer and separated by electrophoresis, and RhoG was identified by immunoblotting.

##### Electron Microscopy

Subcellular platelet morphology was analyzed by transmission electron microscopy. Ultrathin counterstained sections were prepared as described previously (29), imaged with a Tecnai Spirit T12 microscope (FEI), and analyzed with NIH ImageJ 1.46. Granule numbers were quantified manually and expressed as granules/cell/image.

##### Turbidometric Aggregometry and ATP Secretion

Aggregation studies were conducted using aliquots of washed platelets (245 μl, 2 × 10^8^/ml) in a Born lumi-aggregometer (560-VS, Chrono-Log) and stirred at 1000 rpm (37 °C). ATP secretion was measured simultaneously using a luciferin-luciferase assay calibrated with 2 nmol of ATP standards.

##### Platelet Spreading

Static platelet adhesion and spreading assays were performed as described previously (26). Images were analyzed with ImageJ in a blinded fashion.

##### Flow Cytometry

Assays were as described (26). Surface glycoprotein expression was determined in duplicate, and median values were used for calculations. Integrin α_IIb_β_3_ activation and P-selectin expression in response to agonist stimulation were determined simultaneously. Intracellular F-actin levels were estimated using FITC-phalloidin binding to Triton X-100-permeabilized platelets. Analyses were performed using a flow cytometer with proprietary software (FACSCanto II and FACSDiva, BD Biosciences). At least 30,000 platelet-gate events were collected per experiment.

##### Intracellular Calcium Signaling

Changes in cytosolic [Ca^2+^]*_i_* were measured by spectrofluorometry in washed platelets (5 × 10^7^/ml) loaded with the Ca^2+^-sensitive dye Fura-PE3 as described previously (30). Platelets were stimulated (37 °C) with agonists in the absence or presence of inhibitors as indicated, with continuous stirring. Fura-PE3 was excited alternately at 340 and 380 nm, and fluorescence emission was detected at 510 nm. Fluorescence signals were corrected for autofluorescence and calibrated in terms of [Ca^2+^]*_i_*.

##### β-Hexosaminidase Assay

Lysosomal secretion was measured as described (31) with modifications. Briefly, 100-μl aliquots of 10 mm 4-nitrophenyl *N*-acetyl-β-d-glucosaminide in citrate/phosphate buffer (0.2 m Na_2_HPO_4_ and 0.1 m citric acid, pH 4.2) were placed into 96-well plates. Washed platelet aliquots (45 μl, 2 × 10^8^/ml) were stimulated for 10 min at 20 °C as indicated and immediately centrifuged for 5 min at 500 × *g*, and supernatant (6 μl) was added to relevant wells. Tyrode's HEPES buffer was used for blanks. Total β-hexosaminidase activity was measured on repeated snap-frozen/thawed samples. After 18 h of incubation at 37 °C, plates were quenched with 100 μl of 0.12 m NaOH and read at 405 nm (Opsys MR, Dynex Technologies). Blank values were subtracted, and secreted β-hexosaminidase was expressed as a percentage of the total activity.

##### G-LISA® Assays

Plate-based activation assays for Rac and RhoA (Cytoskeleton, Inc.) were conducted following the manufacturer's instructions with modifications for lysate preparation. Briefly, washed platelet aliquots (50 μl, 7.5 × 10^8^/ml) were stimulated as indicated (20 °C) and lysed on ice with equal volumes of 2× lysis buffer, and 50 μl of lysate was added to relevant wells.

##### In Vitro Thrombus Formation

Whole blood flow chamber assays were performed as described previously (32). Blood anticoagulated with sodium citrate, heparin (2 units/ml), and d-phenylalanyl-prolyl-arginyl chloromethyl ketone (40 μm) and labeled with 3,3′-dihexyloxacarbocyanine iodide (1 μm) was flowed at 1000 s^−1^ for 3 min over immobilized collagen (50 μg/ml) in parallel plate perfusion chambers. Phase-contrast and fluorescence images were captured at 2 frames/s with a 40× water dipping objective (BX51WI, Olympus), a Rolera-XR digital camera, and QCapture software (QImaging). Non-adherent cells were removed by flowing Tyrode's HEPES buffer through the chambers. Thirty random images of the collagen-coated surface were collected per experiment, and the surface area covered by platelets was analyzed with ImageJ.

##### Ferric Chloride Carotid Injury Model

*In vivo* thrombus formation assays were performed as described previously (33). Mice were anesthetized with 100 mg/kg ketamine (Vetalar V, Pfizer) and 10 mg/kg xylazine (Rompun, Bayer). Platelets were labeled by intravenous administration of 100 mg/kg DyLight 488-conjugated anti-GPIbβ antibody. Right carotid arteries were exposed, and 15% ferric chloride-soaked filter paper (2 × 1 mm) was placed on the arterial adventitia for 3 min. Time-lapse microscopy of the injury site for 20 min was performed, and images were processed using ImageJ. Background florescence values measured upstream of the injury site were subtracted from thrombus-specific fluorescence, and data are expressed as integrated density values.

##### Tail Bleeding Time

Mice were anesthetized as described above. Using a scalpel, 5 mm of tail was resected from the tip, and the tail was immersed in saline (37 °C). Times from incision to cessation of bleeding and total bleeding time (including re-bleeds) were recorded. The maximum allowable bleeding time was 600 s.

##### Statistics

Unless stated otherwise, the data presented are means ± S.E., with statistical significance determined by Student's *t* test performed using Prism 5.0 (GraphPad Software). *p* < 0.05 was considered significant.

## RESULTS

### 

#### 

##### RhoG Is Expressed and Activated in Human Platelets

**FIGURE 1.**
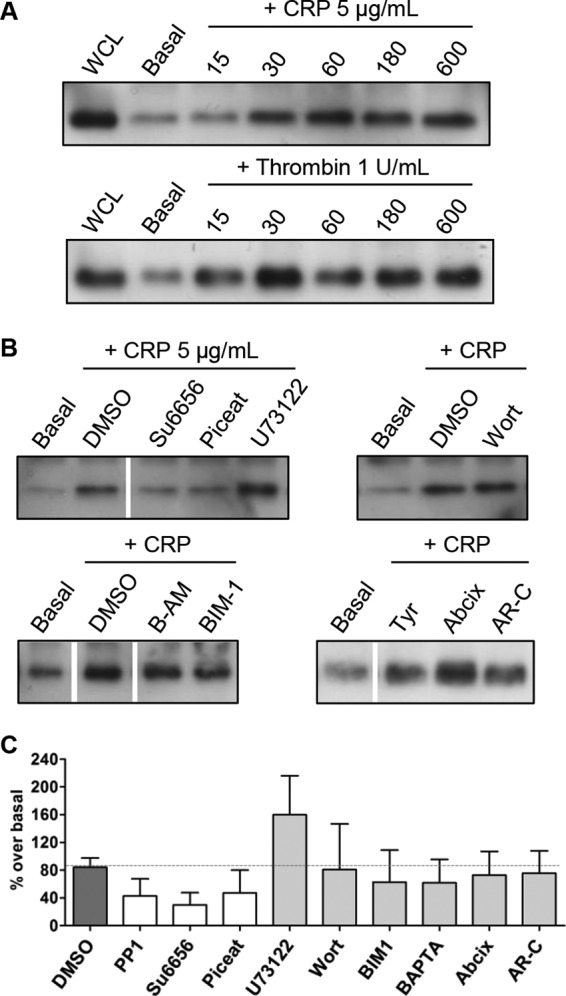
*A*, immunoblotting for RhoG on platelet whole cell lysates demonstrated RhoG expression in human platelets. GST-ELMO fusion protein pulldown assays were then used to assess activation of RhoG in human platelets. The fusion protein interacted specifically with the activated (GTP-bound) form of RhoG. Platelet whole cell lysates (*WCL*) were stimulated with cross-linked CRP or with bovine α-thrombin at the indicated final concentrations, and the time course of RhoG activation was assessed by immunoblotting for RhoG in the pulldown samples. *B*, the GST-ELMO activation assay was then used to evaluate the effect of various pharmacologic inhibitors on RhoG activation in CRP-stimulated human platelets. Inhibitors were incubated with washed platelet suspensions for 10 min prior to stimulation. Compared with the appropriate vehicle control dimethyl sulfoxide (*DMSO*) or Tyrode's HEPES buffer (*Tyr*), RhoG activation was reduced by Su6656 (20 μm) and piceatannol (*Piceat*; 60 μm) but not by U73122 (10 μm), wortmannin (*Wort*; 100 nm), BAPTA-AM (*B-AM*; 10 μm), BIM1 (10 μm), Abciximab (*Abcix*; 10 μg/ml), or AR-C66096 (*AR-C*; 1 μm). This suggests that RhoG activation in human platelets is dependent on Src family kinases and Syk activity and that RhoG activation is independent of PLC, PI3K, or PKC activity and does not require intracellular calcium or signaling downstream of α_IIb_β_3_ or P2Y_12_ receptors. All images are representative of three separate experiments. The *white lines* in the lower immunoblot panels denote gaps between different sections of the same membrane. *C*, the effects of these inhibitors on RhoG activation were quantified by densitometry using ImageJ. *Bars* represent the mean, and *error bars* represent S.E. from at least three membranes/group. The *dotted gray line* represents the mean level of the dimethyl sulfoxide controls.

Immunoblotting confirmed expression of RhoG in human platelets (Fig. 1*A*). GST-ELMO binding assays were used to evaluate RhoG activation in platelets following stimulation with thrombin or CRP. In human platelets, both agonists activated RhoG robustly, but activation in response to thrombin occurred more rapidly than in response to CRP stimulation (Fig. 1*A*). The GPVI signaling pathway is well characterized, so we used pharmacologic inhibitors to investigate which signaling events are required for RhoG activation. In CRP-stimulated platelets, the activation of RhoG was inhibited by the Src inhibitor Su6656 and the Syk inhibitor piceatannol (Fig. 1, *B* and *C*). RhoG activation was not inhibited by U73122, wortmannin, or BIM1, however, showing that PLCγ2, PI3K, and PKC activities are not required. Similarly, intracellular calcium signaling, outside-in signaling through α_IIb_β_3_, and P2Y_12_ signaling are not required because treatment with BAPTA-AM, Abciximab, and the P2Y_12_ antagonist AR-C66096 did not affect RhoG activation. These data suggest that RhoG activation downstream of GPVI is a membrane-proximal event, which likely follows assembly of the LAT signalsome, resulting from Syk activation (34).

##### Hematology Indices and Platelet Ultrastructure Are Normal in RhoG^−/−^ Mice

**FIGURE 2.**
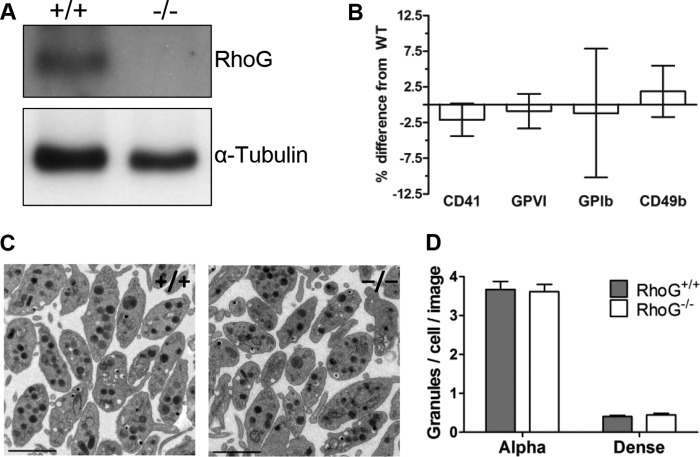
*A*, immunoblotting confirmed RhoG protein expression in RhoG^+/+^ mouse platelets and ablation of RhoG protein expression in platelets from constitutive RhoG knock-out mice (RhoG^−/−^). *B*, flow cytometry was used to evaluate the expression of key platelet surface glycoproteins in RhoG^−/−^ mice. For each experiment, surface expression was determined in duplicate by evaluating the binding of FITC-conjugated monoclonal antibodies. Isotype antibody controls were used to account for nonspecific binding. There were no significant differences between WT and RhoG^−/−^ platelet expression of CD41, GPVI, GPIb, or CD49b. *Bars* represent the mean, and *error bars* represent S.E. (*n* = 10). *C*, to evaluate RhoG^−/−^ platelet ultrastructure, ultrathin sections were imaged with a transmission electron microscope. Subjectively, there was no difference in the subcellular morphology of WT platelets compared with those lacking RhoG. *Scale bars* = 2 μm. *D*, the numbers of platelet α-granules and dense granules were evaluated using transmission electron microscopic sections taken at ×2800 magnification. The total numbers of platelet granules in 10 equivalent-sized fields of view were quantified manually using ImageJ, and granule numbers are expressed as granules/cell/image. *Bars* represent means ± S.E. of three mice/group. There was no significant difference in the numbers of α-granules or dense granules between WT and RhoG^−/−^ platelets.

To study the function of RhoG in platelets, we used a previously reported constitutive RhoG^−/−^ mouse line (25) and confirmed that RhoG protein expression is ablated in platelets from these mice (Fig. 2*A*). Although immune function is abnormal in RhoG^−/−^ mice, lymphocyte numbers are reportedly normal (25). Our hematology data are consistent with that and also show that RhoG mice have normal platelet counts and normal platelet volumes (Table 1). Analyses of platelet surface glycoprotein expression demonstrated normal levels of GPVI, GPIb, and integrins α_IIb_β_3_ and α_2_β_1_ (Fig. 2*B*), whereas transmission electron microscopy images revealed normal ultrastructure in RhoG^−/−^ platelets with normal numbers of α-granules and dense granules (Fig. 2, *C* and *D*).

**TABLE 1:** **Hematology data**

	RhoG^+/+^			RhoG^−/−^			*p*
	Mean	S.E.	*n*	Mean	S.E.	*n*	
Platelets (×10^9^/liter)	731.2	24.25	22	750.8	20.79	22	ns*^a^*
Mean platelet volume (fl)	5.09	0.04	22	5.10	0.03	22	ns
Plateletcrit (%)	0.37	0.01	22	0.38	0.01	22	ns
Leukocytes (×10^9^/liter)	8.43	0.60	22	10.41	0.54	22	ns
Erythrocytes (×10^12^/liter)	9.80	0.10	22	9.82	0.17	22	ns
Hematocrit (%)	46.9	0.46	22	47.5	0.87	22	ns
Mean corpuscular volume (fl)	47.8	0.30	22	48.3	0.32	22	ns

*^a^* ns, not significant.

##### Integrin Activation and Aggregation Are Defective in RhoG^−/−^ Platelets Downstream of GPVI

**FIGURE 3.**
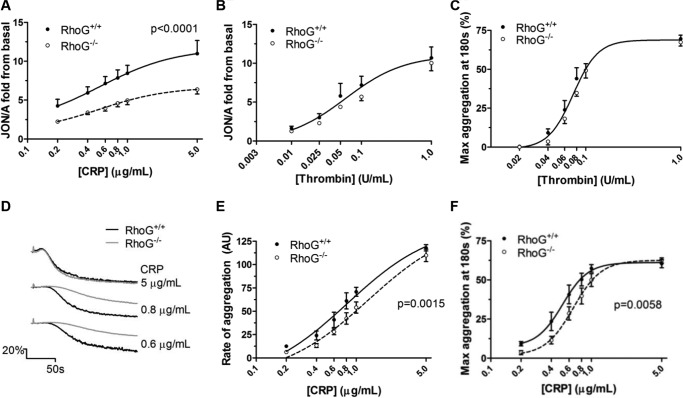
*A*, inside-out signaling to integrin α_IIb_β_3_ activation in WT and RhoG^−/−^ platelets assessed by flow cytometry. Stimulation of platelets with CRP in the presence of 2 mm CaCl_2_ led to dose-dependent increases in JON/A binding, indicating increasing integrin activation. Compared with WT platelets, integrin activation was significantly reduced in RhoG^−/−^ platelets (*p* < 0.0001 by extra sum-of-squares *F*-test). *B*, on stimulation with thrombin, however, there was no significant difference between integrin activation in WT and RhoG^−/−^ platelets. *C*, as with measurements of integrin activation, thrombin-stimulated aggregation was normal in RhoG^−/−^ platelets. *D–F*, the defect in integrin activation downstream of GPVI noted in the RhoG^−/−^ platelets translated into significant reductions (by extra sum-of-squares *F*-test) in the rate and maximum extent of CRP-induced aggregation. Data are presented as means ± S.E. from at least five mice/group.

Activation of the platelet fibrinogen receptor following CRP stimulation was assessed by binding of the activation state-specific antibody JON/A. At all concentrations of CRP evaluated (0.2–5 μg/ml), integrin activation was significantly reduced in RhoG^−/−^ platelets (Fig. 3*A*). In contrast, integrin activation was normal in RhoG^−/−^ platelets when stimulated with thrombin (Fig. 3*B*). Aggregation responses to thrombin were normal in RhoG^−/−^ platelets (Fig. 3*C*). The defect in integrin activation in response to CRP translated into reductions in the rate and maximum extent of aggregation at all concentrations of CRP except maximal (Fig. 3 *D–F*).

##### Investigating the Mechanism of the Integrin Activation and Aggregation Defects

**FIGURE 4.**
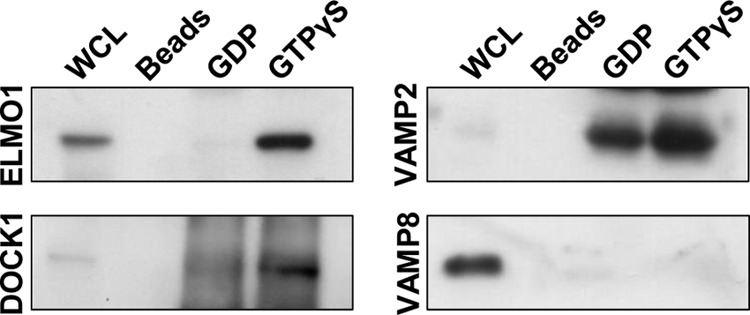
GST-RhoG fusion protein loaded with either GDP or GTPγS was used to pull down potential interacting proteins from human platelet whole cell lysates (*WCL*). Candidate interacting proteins were initially identified by mass spectrometry. Immunoblotting was then used to confirm the specificity of these interactions. Only active (GTPγS-bound) RhoG bound ELMO1 and DOCK1 (DOCK180). VAMP2 interacted with both GDP- and GTPγS-loaded RhoG, but there was greater binding to the active form. RhoG interacted only with certain SNARE proteins, however; for instance, RhoG did not interact with VAMP8 in either the GDP- or GTP-loaded state. GSH-Sepharose beads were used to control for nonspecific binding. None of these proteins bound to the beads themselves.

To explore potential causes of these defects, we first used GST-RhoG pulldown assays to identify RhoG effector proteins in platelets. Three databases of proteins from human platelet lysates that interacted with GST-RhoG were generated by a proteomics approach, compiled from different donors on separate occasions (supplemental Data A–C). Proteins that specifically bound to active (GTPγS-loaded) RhoG were identified and grouped by function (Table 2), and the interaction of selected candidate proteins was confirmed by immunoblotting. Several plausible mechanisms were identified, including activation of the Rac1 guanine nucleotide exchange factor (GEF) proteins ELMO1 and DOCK1 (DOCK180), regulation of the actin cytoskeleton, and interactions with components of the granule secretion machinery. Human platelets express ELMO1 and DOCK1, and RhoG interacted with both proteins when GTP-loaded (Fig. 4). In addition, active RhoG also preferentially interacted with regulators of platelet secretion, including the vesicle SNARE protein VAMP2. This interaction is not universal to all VAMP proteins, however, because RhoG did not bind to VAMP8. We also identified novel interactions with several uncharacterized proteins, although the physiologic significance of this is currently unclear.

**TABLE 2:** **Summary of proteomics data**

UniProt accession no.	Protein identified	Protein type/function	Mean score	Mean coverage	Mean no. peptide spectrum matches	Mean no. peptides	*N* × identified (maximum of 3)
**Actin cytoskeleton regulators**							
Q9H7D0	DOCK5	GEF for RhoA and Rac	35	2.2	3	2	3
Q92556	ELMO1	Rac1 GEF component	34	8.4	6	3	3
P19105	Myosin regulatory light chain 12A	Actin cytoskeletal motor protein	10	8	2	1	2
P05109	Protein S100-A8	Calcium-binding protein; CapZ-interacting protein	6.6	11.8	2	1	2
P48059	LIMS1	Actin cytoskeleton adapter	4.6	3.7	2	1	2
Q96BY6	DOCK10	Rho GTPase GEF	64.2	0.4	1	1	1
Q14185	DOCK1	Rac1 GEF component	6.72	1.1	1	1	1

**Microtubule network regulators**							
Q15058	KIF14	Microtubule motor protein	33.1	1.9	1	1	3
Q9Y6G9	Dynein light chain 1	Microtubule motor for vesicles and organelles	24.5	72.3	5	2	1
Q8IWC1	MAP7 domain-containing protein 3	Microtubule-associated protein	11.1	2.9	1	1	1
Q6P597	Kinesin light chain 2	Microtubule motor protein	7.3	13.3	1	1	1
Q92845	Kinesin-associated protein 3	Microtubule motor protein	2.8	4.4	1	1	1

**SNARE complex regulators**							
O94812	BAI1-associated protein3 (BAP3)	Calcium-sensitive synaptotagmin homolog	20.5	13.1	3	1	2
P37840	α-Synuclein	SNARE complex assembly promoter	13.2	38.8	4	2	1
P63027	Vesicle-associated membrane protein 2	v-SNARE	4.3	25	1	1	1
O15155	BET1 homolog	t-SNARE; binds syntaxin	4.5	15.3	1	1	1
O94910	Latrophilin-1	Membrane protein; binds syntaxin	3.4	13.6	1	1	1

**Uncharacterized proteins**							
Q96MK2	FAM65C	Uncharacterized protein	55.9	7.6	12	5	3
A6NFQ2	FAM115C	Uncharacterized protein	18.1	3.9	1	1	2
E9PR74	Uncharacterized protein	Uncharacterized protein	27.9	8.8	2	1	2
F2Z353	Uncharacterized protein	Uncharacterized protein	21.3	15.3	1	1	2
Q86TV6	TTC7B protein	Uncharacterized protein	8	8.6	1	1	2

**FIGURE 5.**
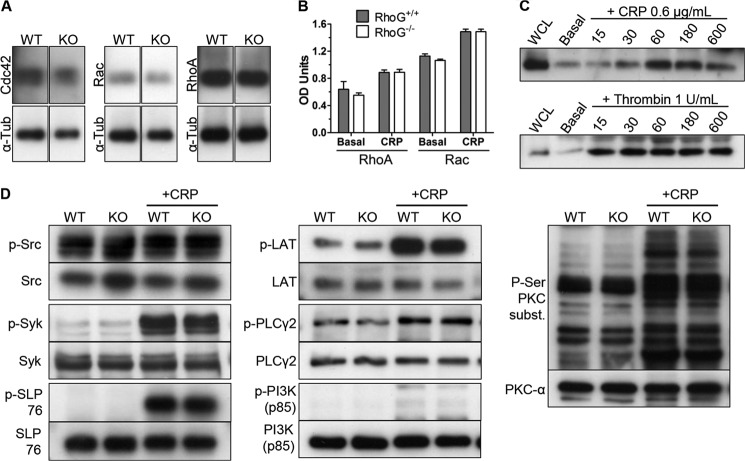
*A*, immunoblotting for other Rho family GTPases in WT and RhoG^−/−^ platelets demonstrated that there was no reduction in expression of Cdc42, Rac, or RhoA in RhoG^−/−^ platelets. *KO*, knock-out; α*-Tub*, α-tubulin. *B*, plate-based G-LISA assays were used to evaluate the activation of RhoA and Rac in WT and RhoG^−/−^ platelets in response to CRP. Stimulation of washed platelets with CRP (0.6 μg/ml) activated Rac and RhoA, but no differences in the activity of either GTPase was noted in basal or post-stimulation samples from RhoG^−/−^ platelets compared with WT platelets, suggesting that as in lymphocytes, RhoG does not act via Rac in platelets. Data are presented as means ± S.E. of three mice/group for each condition. *C*, the activation of RhoG in mouse platelets in response to CRP and thrombin stimulation was assessed using a GST-ELMO pulldown assay. In mouse platelets, RhoG was activated by both CRP and thrombin, although at the concentrations used, RhoG was activated more slowly in response to CRP compared with thrombin. *WCL*, whole cell lysate. *D*, the role of RhoG in the tyrosine kinase signaling pathway downstream of GPVI was evaluated using phosphorylation-specific antibodies. Washed platelets from WT and RhoG^−/−^ mice in basal states and following stimulation with 0.6 μg/ml CRP for 60 s were lysed and prepared for immunoblotting. Phosphorylation of Src family kinases (Tyr-416), Syk (Tyr-525/Tyr-526), SLP76 (Tyr-128), PI3K p85 (Tyr-458), LAT (Tyr-191), PLCγ2 (Tyr-759), and the substrates of PKC (phospho-Ser) was assessed. Immunoblots using antibodies against the non-phosphorylated forms were used as loading controls. No change in Src family kinase phosphorylation status was noted, but for the other kinases assessed, phosphorylation increased upon CRP stimulation. No differences between the WT and RhoG^−/−^ platelets were seen in basal or stimulated samples, however. Immunoblots are representative of at least three separate experiments.

Because activated RhoG interacts with the Rac1 GEF DOCK1, we speculated that the defects in RhoG^−/−^ platelets might be due to altered signaling by the downstream GTPase Rac or Cdc42 (18, 35). We first confirmed that expression of RhoA, Rac, and Cdc42 was unaffected by RhoG expression (Fig. 5*A*). Then, using plate-based (G-LISA) activation assays, we assessed Rac and RhoA activation in RhoG^−/−^ platelets stimulated by CRP (0.6 μg/ml). Both basal and post-stimulation levels of activated Rac and RhoA in RhoG^−/−^ platelets were equivalent to those in WT platelets, showing that RhoG does not control activation of these small G-proteins in platelets downstream of GPVI (Fig. 5*B*).

The difference between the responses of RhoG^−/−^ platelets to CRP and thrombin stimulation was interesting, and we wondered if RhoG is actually activated by thrombin stimulation of mouse platelets. Activation of RhoG was therefore assayed in WT platelets in response to 1 unit/ml thrombin or a concentration of CRP (0.6 μg/ml) that exposed aggregation abnormalities in RhoG^−/−^ platelets. This showed that both thrombin and CRP activated RhoG in mouse platelets (Fig. 5*C*). Although this did not provide an explanation for the difference between the effects of CRP and thrombin stimulation, it clearly demonstrated that lack of activation of RhoG was not the cause of the defects seen in RhoG^−/−^ platelets following CRP stimulation.

To investigate whether RhoG might be exerting its function by regulating the GPVI tyrosine kinase signaling pathway, we assessed the phosphorylation of key members of this signaling cascade following CRP stimulation. We found no differences in the phosphorylation status in either basal or activated states between the WT and RhoG^−/−^ platelets (Fig. 5*D*).

**FIGURE 6.**
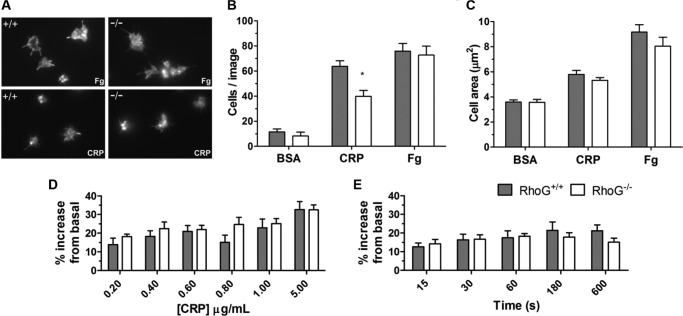
*A*, the ability of RhoG^−/−^ platelets to adhere to and spread on agonist surfaces was evaluated under static conditions. Glass coverslips were coated with fatty acid-free BSA, CRP, or fibrinogen (*Fg*). Aliquots of washed platelets were applied to the coverslips and allowed to interact with the surface for 20 min. After the allotted time, unbound cells were gently removed by washing with buffer, and bound cells were fixed with 4% paraformaldehyde. Adherent platelets were then permeabilized, and their F-actin content was stained with TRITC-conjugated phalloidin to allow image capture by fluorescence microscopy. The numbers of cells adherent after 20 min (*B*) and the mean cell surface area/platelet (*C*) were measured using ImageJ. *, *p* < 0.05 for the comparison between matched columns. BSA was used as a control, with minimal adhesion and essentially no spreading occurring on this surface. Significantly fewer (*p* = 0.0375) RhoG^−/−^ platelets adhered to CRP compared with WT platelets. Adherence to fibrinogen was normal, however. There was no significant difference in the surface area of RhoG^−/−^ platelets spread on CRP or fibrinogen compared with WT platelets. Data are presented as means ± S.E. of at least four mice/group. *D*, generation of F-actin was assessed using a FITC-phalloidin-based flow cytometry assay. Washed platelets were stimulated with CRP at the indicated final concentrations (*D*) or with 0.6 μg/ml CRP for the indicated time periods (*E*), fixed, permeabilized, and stained with FITC-conjugated phalloidin. The FITC signal is proportional to the F-actin content. No significant differences in F-actin generation were noted in RhoG^−/−^ platelets in response to a range of CRP concentrations or over a time course at a given concentration. This suggests that the functional defects noted in platelets lacking RhoG are not due to abnormal actin turnover. Data are presented as means ± S.E. of six mice/group.

We next evaluated the ability of RhoG^−/−^ platelets to adhere to and spread on agonist surfaces. After 20 min, the number of RhoG^−/−^ platelets stably adhered to CRP was significantly reduced, but there was no defect in cell spreading on CRP (Fig. 6, *A–C*). Adhesion to and spreading on fibrinogen-coated surfaces was normal in RhoG^−/−^ platelets, suggesting that α_IIb_β_3_ outside-in signaling is unaffected by loss of RhoG. Actin remodeling is integral to Rho GTPase function in platelets, and RhoG is known to regulate the actin cytoskeleton in lymphocytes (12, 20). To investigate if this might be contributing to the functional defects seen, we evaluated actin polymerization in RhoG^−/−^ platelets in response to CRP stimulation using a FITC-phalloidin binding assay. No significant differences between WT and RhoG^−/−^ platelets were seen (Fig. 6, *D* and *E*).

##### RhoG^−/−^ Platelets Have Reduced Granule Secretion, Which Explains the Integrin Activation and Aggregation Defects Following CRP Stimulation

**FIGURE 7.**
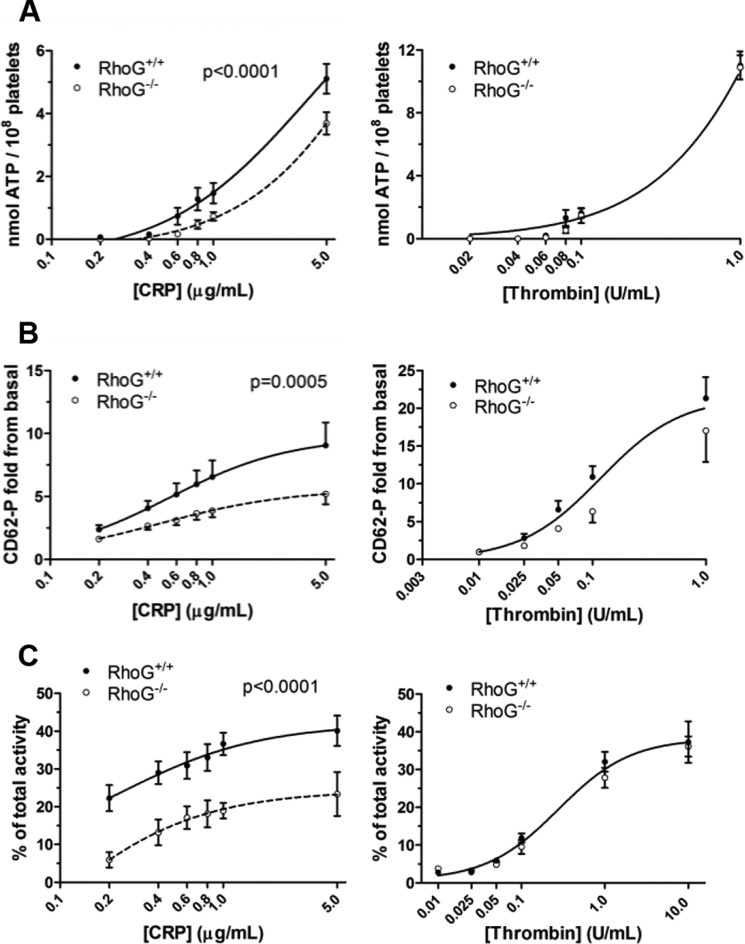
Secretion from dense granules, α-granules, and lysosomes was assessed in RhoG^−/−^ platelets following stimulation with CRP or thrombin. *A*, dense granule secretion, measured by luminescence during aggregometry, was significantly reduced when CRP was the agonist but not when thrombin was used to induce aggregation. *B*, this pattern was repeated with α-granule secretion as measured by cell surface P-selectin (CD62P) expression. CRP stimulations for P-selectin expression were performed in the presence of 2 mm CaCl_2_. Lysosomal secretion was measured using a plate-based β-hexosaminidase assay, and values are expressed as a percentage of the total β-hexosaminidase activity. *C*, lysosomal secretion in response to CRP stimulation (in the presence of 2 mm CaCl_2_) was significantly reduced in RhoG^−/−^ platelets but was normal in response to thrombin stimulation. Data are presented as means ± S.E. from at least four mice/group.

Because RhoG interacts with the SNARE protein VAMP2, we investigated whether granule secretion is involved in the integrin activation and aggregation defects seen in RhoG^−/−^ platelets. On examining secretion from α-granules, dense granules, and lysosomes, we found defects in secretion from all three granule types in RhoG^−/−^ platelets. As elsewhere, these reductions occurred following CRP stimulation, whereas RhoG^−/−^ platelet secretion following thrombin stimulation was normal (Fig. 7, *A–C*).

**FIGURE 8.**
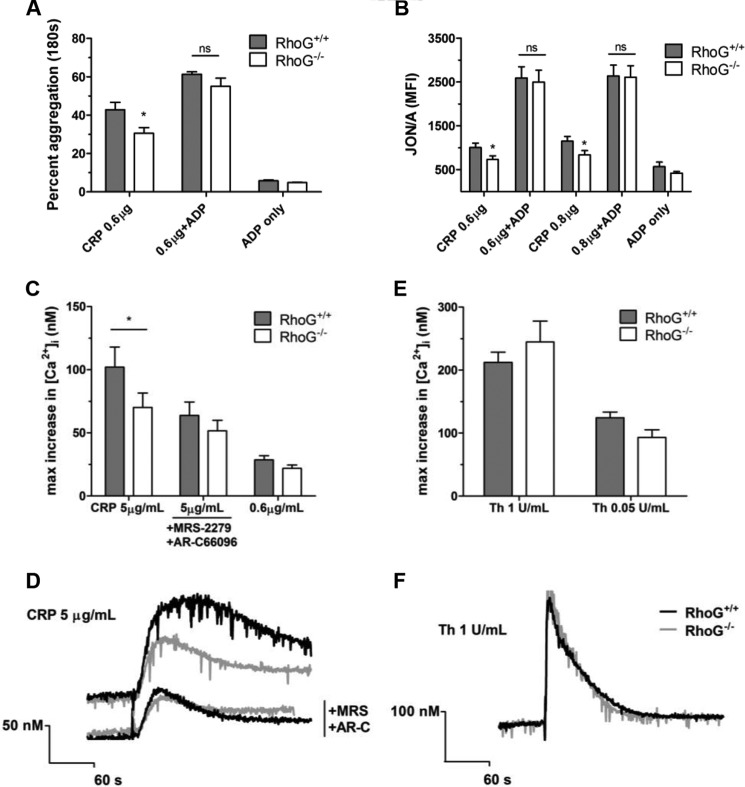
To test whether the defects noted in RhoG^−/−^ platelet function downstream of GPVI were due to reduced dense granule secretion, we co-stimulated platelets with CRP and ADP (10 μm). *A*, in the aggregometer, ADP in addition to CRP augmented the WT and RhoG^−/−^ responses and rescued the defect seen in the RhoG^−/−^ platelets. *B*, similarly, when ADP was used to co-stimulate platelets with CRP (in the presence of 2 mm CaCl_2_), the defective integrin activation in RhoG^−/−^ platelets was restored to WT levels. *C*, to test whether these phenomena were due to defective feedback signaling downstream of P2Y receptors, we evaluated the intracellular calcium fluxes in RhoG^−/−^ platelets in the presence or absence of P2Y receptor-mediated signals. *, *p* < 0.05 for the comparison between matched columns; *ns*, no significant difference. When P2Y receptor signaling was present, there were reduced intracellular calcium fluxes observed in RhoG^−/−^ platelets following CRP (5 μg/ml) stimulation (*p* = 0.0110) (*C* and representative traces in *D*). Inhibitors MRS-2279 (*MRS*; 10 μm) and AR-C66096 (*AR-C*; 1 μm) were then used to ablate P2Y_1_ and P2Y_12_ signaling, respectively. In the presence of these inhibitors, the difference between WT and RhoG^−/−^ platelet intracellular calcium fluxes was abolished, suggesting that the differences observed between WT and RhoG^−/−^ calcium signals relate to differences in P2Y receptor signaling. No differences between WT and RhoG^−/−^ intracellular calcium fluxes were noted following thrombin (*Th*; 1 unit/ml) stimulation (*E* and representative traces in *F*). Data are presented as means ± S.E. from at least four mice/group.

Autocrine and paracrine signaling through P2Y_12_ augments platelet aggregation and inside-out signaling occurs between P2Y_12_ and α_IIb_β_3_. We speculated therefore that the defects seen in RhoG^−/−^ platelet CRP responses might be due to reduced positive feedback resulting from reduced dense granule ADP release. We tested this by co-stimulating RhoG^−/−^ platelets with CRP and ADP. Co-stimulation of RhoG^−/−^ platelets with CRP (0.6–0.8 μg/ml) and ADP (10 μm) augmented the integrin activation and aggregation responses of both WT and RhoG^−/−^ platelets and critically normalized the responses of the RhoG^−/−^ platelets (Fig. 8, *A* and *B*).

These findings support the dense granule defect as the primary lesion in RhoG^−/−^ platelets, leading to reduced feedback through P2Y_12_. To confirm this, we stimulated platelets with CRP and evaluated the changes in intracellular calcium. CRP stimulation increased intracellular calcium concentrations but was attenuated in RhoG^−/−^ platelets (Fig. 8, *C* and *D*). Because P2Y receptor activation leads to PLCβ activation via G_q_ and G_i2_, which augments and sustains the calcium signal (36), we also evaluated the intracellular calcium signals in the presence of the P2Y_1_ and P2Y_12_ antagonists MRS-2279 and AR-C66096. In the absence of P2Y signaling, there were no significant differences in intracellular calcium fluxes between WT and RhoG^−/−^ platelets (Fig. 8, *C* and *D*), confirming that when ADP signaling is absent, there is no additional abnormality in calcium signaling in RhoG^−/−^ platelets. No abnormalities in calcium signaling following thrombin stimulation were observed in RhoG^−/−^ platelets (Fig. 8, *E* and *F*).

##### RhoG Is Required for Thrombus Formation in Vitro and in Vivo

**FIGURE 9.**
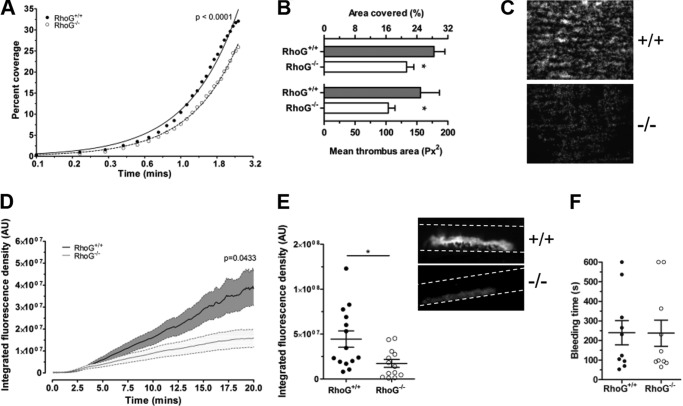
The potential physiologic consequences of the defects in GPVI-mediated granule secretion noted in RhoG^−/−^ platelets were assessed. Whole blood anticoagulated with citrate, heparin, and d-phenylalanyl-prolyl-arginyl chloromethyl ketone was flowed over fibrillar collagen at 1000 s^−1^ using an *in vitro* parallel plate flow chamber system. *A*, the change in surface area covered by RhoG^−/−^ platelets over time was significantly lower (*p* < 0.0001 by extra sum-of-squares *F*-test) compared with WT platelets (*n* = 10). After washing to remove non-stably adhered cells, the surface area covered by RhoG^−/−^ platelets was significantly lower compared with WT platelets (*p* = 0.0101). This was principally due to lower mean thrombus size in RhoG^−/−^ platelets (*p* = 0.0346; *n* = 10) (*B* and representative images in *C*). We then assessed the ability of RhoG^−/−^ mice to form thrombi *in vivo*. Using a ferric chloride-induced carotid artery injury model, we measured the accumulation of thrombus at the site of injury. *D*, mice lacking RhoG formed thrombi at a significantly lower rate compared with WT platelets (*p* = 0.0433 by two-way analysis of variance; *n* = 14), resulting in significantly smaller thrombi 20 min after injury (*E* and *inset* representative images). *, *p* < 0.05 for the comparison between matched columns. *F*, tail bleeding times were not significantly different in RhoG^−/−^ mice (*n* = 10).

To evaluate the physiologic relevance of the dense granule secretion defects seen in washed platelet preparations, we tested the ability of RhoG^−/−^ platelets to adhere to fibrillar collagen under arterial shear. In whole blood under non-coagulating conditions, the accumulation of RhoG^−/−^ platelets over time was significantly lower compared with WT platelets (Fig. 9*A*). After removal of non-stably adhered platelets, the surface area covered by RhoG^−/−^ platelets was significantly lower compared with WT platelets (Fig. 9, *B* and *C*), consistent with the reductions in RhoG^−/−^ platelet responses to CRP.

Because *in vitro* flow assays suggested that RhoG was relevant to platelet physiology under shear, we tested the ability of RhoG^−/−^ mice to form thrombi *in vivo*. Following ferric chloride injury of the carotid artery, RhoG^−/−^ mice formed thrombi at significantly lower rates compared with WT mice (Fig. 9*D*), and the thrombi 20 min post-injury were significantly smaller in RhoG^−/−^ mice (Fig. 9*E*). These findings prompted us to assess the hemostatic potential of RhoG^−/−^ mice. Tail bleeding times were normal in RhoG^−/−^ mice (Fig. 9*F*), suggesting that RhoG deletion does not lead to a hemostatic abnormality.

## DISCUSSION

In this study, we have demonstrated that RhoG controls platelet granule secretion following CRP stimulation, which reduces the positive autocrine and paracrine feedback loops that exist to accelerate platelet activation. We have shown that the contribution of RhoG to dense granule secretion is particularly important in this context because co-stimulation with ADP ameliorates the integrin activation and aggregation defects.

Platelet ADP secretion is necessary for stabilizing thrombi under shear conditions. Our analyses of RhoG platelets forming thrombi on collagen suggest that the defect in RhoG^−/−^ platelet function relates less to the initial interactions with the surface but rather to the recruitment of additional platelets to growing thrombi. This reduction in secondary recruitment is consistent with the secretion defects identified in other assays. Furthermore, the secretion defect downstream of GPVI leads to a reduction in the ability of RhoG^−/−^ mice to form arterial thrombi. The diminished thrombus formation *in vivo* following ferric chloride injury of the carotid artery suggests that granule secretion controlled by RhoG is an important contributor under these conditions. It should be noted that the role played by collagen-GPVI interactions in the ferric chloride model is contested. Several studies have shown that mice immunodepleted of GPVI or without GPVI surface expression (37–39) have impaired thrombus formation in response to ferric chloride, strongly suggesting that the model reflects collagen-GPVI interactions. Others have reported normal thrombus formation in GPVI-deficient mice, however (40). Methodological differences may underlie these divergent data (41). In our study, the defective secretion downstream of GPVI due to the absence of RhoG was clearly sufficient to impair thrombus formation, suggesting that our model is sensitive to collagen-induced platelet secretion.

This thrombotic defect is not paralleled by a hemostatic abnormality, however, because RhoG^−/−^ mouse tail bleeding times were normal, and there was no evidence of a bleeding propensity. It is possible that intact protease-activated receptor-mediated signaling in RhoG^−/−^ mice is sufficient to compensate for the abnormal granule secretion after collagen stimulation or that the tail bleeding model we employed is insensitive to collagen pathway defects (42).

We have demonstrated that deletion of RhoG impairs platelet secretion, and we suggest that this translates into a platelet adhesion defect in whole blood flowed over immobilized collagen *ex vivo*. By extension, we also suggest that this platelet defect is sufficient to reduce thrombus formation following ferric chloride carotid injury. It must be noted, however, that in the constitutive knock-out mouse strain we used, RhoG has also been deleted from leukocytes and from endothelial cells, both of which are known to contribute to thrombosis in this *in vivo* model (41, 43). Thus we cannot exclude that RhoG deletion in these tissues contributed to the thrombosis defect identified, particularly because RhoG deletion causes leukocyte dysfunction (20). Evaluation of a platelet-selective gene deletion model such as a PF4-driven conditional knock-out mouse will be necessary to fully define the role of RhoG in thrombus formation *in vivo*.

We found a consistent difference between the responses of RhoG^−/−^ platelets to CRP and thrombin stimulation, which is most likely explained by the divergent signaling pathways engaged by GPVI and the protease-activated receptors. Stimulation of GPVI activates Syk, leading to the assembly of the LAT signalsome, consisting of various protein and lipid kinases and adapter proteins. Syk activates PLCγ2, which then activates PKC and increases intracellular calcium. In contrast, protease-activated receptors couple to G_q_ and G_13_. Signaling through G_13_ activates RhoA and hence ROCK (Rho-associated protein kinase), whereas G_q_ signals through PLCβ to activate PKC and to increase intracellular calcium.

The GEF for RhoG in platelets is unknown. The LAT signalsome that assembles after GPVI activation includes the GEFs Vav1 and Vav3. In fibroblasts and T-lymphocytes, Vav proteins act as GEFs for RhoG (20, 44). Interestingly, Vav1/Vav3^−/−^ mice have a similar (although not identical) phenotype to the RhoG^−/−^ mice presented here, including reduced platelet aggregation responses to CRP but normal aggregation responses to thrombin. Similarly, Vav1/Vav3^−/−^ platelets also adhere poorly to collagen under shear. In contrast to RhoG^−/−^ platelets, PLCγ2 phosphorylation is reduced in response to CRP in Vav1/Vav3^−/−^ mice. This suggests that Vav is involved in PLCγ2 phosphorylation and that RhoG lies downstream of this event, consistent with RhoG activation being dependent on Vav when platelets are stimulated by CRP. Although Vav1 is phosphorylated in platelets by thrombin stimulation (45), the phenotype of Vav1/Vav3^−/−^ mice suggests that Vav proteins do not mediate platelet function following protease-activated receptor activation. In turn, this may explain why RhoG is not required following thrombin stimulation of platelets. The finding that PLCγ2 phosphorylation is unaffected by RhoG expression suggests that two signaling pathways may follow LAT signalsome assembly: one mediated by PLCγ2 and an alternative route linking RhoG to granule secretion independent of intracellular calcium or PKC.

A possible alternative to Vav is Trio, a Rho GEF that can mediate activation of Rac1, RhoG, and RhoA (46). Trio is expressed at the transcript and protein levels in platelets, although there are no reports of its function to date (16, 17). Recently, it has been demonstrated in neuroblastoma cells that Trio is tyrosine-phosphorylated by Fyn and that this phosphorylation enhances Rac activation (47). It is therefore plausible that Fyn phosphorylation of Trio might accelerate RhoG activation downstream of GPVI in platelets.

Using a proteomics approach, we explored the interacting partners of RhoG in platelets. We demonstrated that active RhoG specifically interacts with both ELMO1 and DOCK1 in human platelet lysates. Such interactions have been previously reported in other cell types, and the complex of RhoG, ELMO1, and DOCK1 acts as a GEF for Rac (21). This raised the possibility that RhoG might activate Rac in platelets, which was of potential importance because of the essential roles played by Rac in platelets. We found no evidence of a defect in Rac activation in the absence of RhoG, however, suggesting that Rac activation downstream of GPVI can occur via RhoG-independent routes, such as through Vav.

Regulation of granule secretion by Rho GTPases can occur through actin cytoskeletal manipulation, granule biogenesis, and intracellular calcium signaling regulation (15, 48, 49), but we have shown that none of these are responsible for the phenotype in RhoG^−/−^ platelets. Platelet granule secretion is highly orchestrated and depends on the assembling of vesicle- and membrane-associated docking protein complexes to facilitate membrane engagement and fusion (50). Our proteomics work provides evidence for direct links between active RhoG and regulators of these granule secretion events, including interactions with regulators of the actin cytoskeleton and the microtubule network, both of which facilitate platelet granule release (51, 52). Thus, such interactions link RhoG to granule secretion. In addition, we also identified specific interactions between active RhoG and the SNARE complex regulator α-synuclein, the Munc13 homology domain-containing protein BAP3, and both vesicle and target SNARE proteins. Although these interactions are not definitive evidence of the mechanism by which RhoG controls platelet secretion, they suggest how RhoG might regulate platelet granule release. These interactions are an important avenue for future study because similar connections have been described for Cdc42 and syntaxin (53).

In summary, we used constitutive RhoG^−/−^ mice to identify the function of RhoG in platelets and have shown this GTPase to be an important regulator of platelet granule secretion. In platelets, RhoG is required for the release of granule cargoes, including ADP, that provide stimulatory autocrine and paracrine feedback signals to augment and maintain thrombi under shear. The potential contribution of RhoG-regulated platelet secretion to atherogenesis or inflammatory diseases is intriguing and warrants further investigation.

## Footnotes

3 The abbreviations used are:

GP: glycoprotein
CRP: collagen-related peptide
PLCγ2: phospholipase Cγ2
BAPTA-AM: 1,2-bis(2-aminophenoxy)ethane-*N*,*N*,*N*′,*N*′-tetraacetic acid tetrakis(acetoxymethyl ester)
GTPγS: guanosine 5′-*O*-(thiotriphosphate)
GEF: guanine nucleotide exchange factor
TRITC: tetramethylrhodamine isothiocyanate.
